# Assessing Risk of Bias in Randomized Controlled Trials for Autism Spectrum Disorder

**DOI:** 10.3389/fpsyt.2017.00265

**Published:** 2017-11-29

**Authors:** Paola Matiko Martins Okuda, Cheryl Klaiman, Jessica Bradshaw, Morganne Reid, Hugo Cogo-Moreira

**Affiliations:** ^1^Department of Psychiatry, Universidade Federal de São Paulo, São Paulo, Brazil; ^2^Marcus Autism Center, Children’s Healthcare of Atlanta, Atlanta, GA, United States; ^3^School of Medicine, Emory University, Atlanta, GA, United States

**Keywords:** autism spectrum disorder, risk of bias, confirmatory factor analysis, meta-analysis, randomized controlled trials

## Abstract

**Aim:**

To determine construct validity and reliability indicators of the Cochrane risk of bias (RoB) tool in the context of randomized clinical trials (RCTs) for autism spectrum disorder (ASD).

**Methods:**

Confirmatory factor analysis was used to evaluate a unidimensional model consisting of 9 RoB categorical indicators evaluated across 94 RCTs addressing interventions for ASD.

**Results:**

Only five of the nine original RoB items returned good fit indices and so were retained in the analysis. Only one of this five had very high factor loadings. The remaining four indicators had more measurement error than common variance with the RoB latent factor. Together, the five indicators showed poor reliability (ω = 0.687; 95% CI: 0.613–0.761).

**Conclusion:**

Although the Cochrane model of RoB for ASD exhibited good fit indices, the majorities of the items have more residual variance than common variance and, therefore, did not adequately capture the RoB in ASD intervention trials.

## Introduction

Autism spectrum disorder (ASD) is a neurodevelopmental disorder characterized by impairments in reciprocal social interaction and stereotyped and repetitive behaviors. Several empirically supported behavioral interventions have been shown to improve core features of ASD, including social communication [e.g., Ref. (1–4)]. Psychopharmacological treatments have been shown to decrease secondary symptoms, such as aggression, irritability, and hyperactivity [e.g., Ref. (5)]. As diagnoses are being made at younger ages and as the prevalence of ASD increases, research has increasingly focused on the development of cost-effective, community viable interventions. This is especially relevant as resources are limited and as clinicians aim to effectively and efficiently guide families in making treatment decisions. Information on what treatments are truly effective for which individuals is invaluable. Randomized clinical trials (RCTs) are considered the “gold standard” for determining intervention effectiveness and efficacy. Hundreds of RCTs are published every year, making it nearly impossible for practicing professionals to keep abreast of the effectiveness literature. In addition, RCT results may often contradict one another, and though some studies are well designed, others may be poorly designed and executed.

One way to aggregate these results is *via* systematic reviews and meta-analyses, in which different strategies have been employed to identify and evaluate the risk of bias (RoB). The most widely used strategy is the Cochrane Risk of Bias Tool, developed by the Cochrane Collaboration (6). To assess RoB in RCTs, they recommend the following indicators/items (called *domains* by the authors): random sequence generation (RSG), allocation concealment, participant and personnel blinding, outcome assessment blinding, incomplete outcome data, selective reporting, and other sources of bias, including important circumstance-specific concerns about bias that are not addressed in the other domains.

According to the Cochrane Risk of Bias Tool (6), each indicator should be evaluated by the systematic review’s authors on a scale with three ordinal categories of response: *low risk, unclear risk*, or *high RoB*. Issues regarding the Cochrane tool have emerged due to concerns of reliability. For example, some items lack agreement among authors (45% of the judgments of risk differed), especially for particular items (e.g., blinding, selective reporting). As such, there are large discrepancies in how RoB is being evaluated (7).

The first study in the literature evaluating the construct validity of RoB was recently conducted by Rodrigues-Tartari et al. (8). In a review of RCTs evaluating methylphenidate for ADHD, the authors found that the Cochrane Risk of Bias Tool has poor evidence of construct validity and was an unreliable instrument (ω = 0.642) for the RCTs under review. The authors concluded, however, that such a negative finding might be a context-specific result of sampling 184 primary RCTs.

Due to the prevalence and increased focus of RCTs and systematic reviews covering different types of interventions for ASD, it is necessary to critically evaluate the way that effectiveness and efficacy have been evaluated. Here, we aim to investigate the construct validity of the Cochrane Risk of Bias Tool in the context of RCTs for ASD.

## Method

This research was approved by the Ethics Committee of Research of the Federal University of Sao Paulo (UNIFESP).

Meta-analyses specifically focusing on interventions for ASD were selected. Interventions with other types of developmental disorders were excluded. Ten intervention systematic Cochrane meta-analyses were considered in this study, consisting of 94 original RCTs. The 10 meta-analyses included were Fletcher-Watson, et al. (9), Geretsegger et al. (10), Hurwitz et al. (11), James et al. (12), Oono et al. (13), Reichow et al. (14), Reichow et al. (15), Sinha et al. (16), Williams et al. (17), and Williams et al. (18). The total number of studies within each meta-analysis varied from 1 to 21 (mean of 9 per study, totaling 94 primary RCTs). The meta-analyses were conducted from 2010 to 2015. The studies within the meta-analyses were conducted from 1989 to 2013.

For each included RCT, data extractors (i.e., authors of the systematic reviews) independently evaluated all RoB domains (the items). As such, for this analysis, data were provided by the *RoB summary* in each included study and not determined by the present study’s authors. Each bias domain was detailed in the meta-analysis using an ordinal rating scale consisting of the following three categories: low RoB, uncertain RoB, or high RoB, according to the Cochrane guidelines. Data on the level of agreement between the data extractors (authors of the systematic reviews) across the items are not presented because any disagreements were resolved by discussion and negotiation with a third author, as per best practices. Together with the seven commonly used RoB, based on research by Reichow et al. (15), additional indicators were occasionally used—“*Baseline Measurements (BM)*” and “*Protection against contamination (PAC)*.”

Though the systematic reviews cited above commonly use only the original seven items of bias (excluding BM and PAC), some used the additional indicators (for a total of 9). For these analyses, we consider all reported items; thus, if a given systematic review did not have the additional two items (BM and PAC), they were considered as missing values. Underlying these nine ordinal items an *a priori* latent attribute called RoB was specified. Table 1 depicts the name and code for each risk of bias item included in the analysis.

**Table 1:** Nine items of the “risk of bias” (RoB) factor.

RoB items	Code
Random sequence generation (sequence bias)	RSG
Allocation concealment (selection bias)	AC
Incomplete outcome data (attrition bias)	IOD
Blinding of participants and personnel (performance bias)	BPP
Blind of outcome assessment (detection bias)	BOA
Selective reporting (reporting bias)	SR
Other bias	OB
Baseline measurements	BM
Protection against contamination	PAC

### Statistical Analysis

Confirmatory factor analysis (CFA) was used to evaluate (a) the goodness of fit of the measurement model proposed by the Cochrane Collaboration and (b) the strength of the correlation between the items and the overall RoB factor. In this way, the factor loading is a correlation between the observed categorical item and the latent measure (RoB) where the higher the correlation, the lower the items’ residual variance. As such, the lower the residual variance, the higher the reliability index of each RoB’s indicator. CFA is commonly applied to provide construct validity to tests, scales, questionnaires, and batteries. Typically, these tools assess human attributes such as psychopathology (i.e., depression, anxiety), skills (academic skills), health (well-being, happiness), and other latent measures (i.e., theoretical constructs underlying the test which are not directly observed). Similarly, though less frequently, these techniques are applied to measure non-human attributes in the healthcare field. To the best of our knowledge, there is only one study using CFA to provide construct validity of RoB (8). This study is thus innovative in the application of CFA to RoB in the field of ASD.

The unidimensional model (i.e., a model in which all the RoB items load onto a single latent attribute) was chosen for two primary reasons. First, a reduced number of dimensions may leave the model with a parsimonious solution. This would create the simplest model with the least number of assumptions and variables but with greatest explanatory power in terms of interpretability of the measurement model (19). Second, there are no clear descriptions by Cochrane Collaboration about how the indicators should be grouped to form subfactors to generate a multidimensional solution.

Several fit indices were used to evaluate the RoB model including: chi-square (χ^2^), confirmatory fit indices (CFI), the Tucker–Lewis index (TLI), and root mean square error approximation (RMSEA). The following cutoff criteria were used to determine a good model of fit: a non-statistically significant chi-square *p*-value (>0.05), a RMSEA near or less than 0.06, and CFI and TLI near or greater than 0.95 (20). The weighted least square using a diagonal weight matrix with SEs and mean- and variance-adjusted (WLSMV) estimator was used (21) because the observed indicators (i.e., RoB items) are ordinal [i.e., a categorical item with three possible answers (low, unclear, and high RoB)]. Due to the complex sampling structure (i.e., 94 clinical trials within 10 systematic reviews), SEs were computed by a sandwich estimator and a chi-square test of the model fit which took into account the non-independence of observations. For more detail and discussion about such implementation, see Ref. (22, 23). The adopted statistical significance level was 0.05. All analyses were run using Mplus 8.0 (24).

As the precision of the set of items used to measure the continuous latent attribute RoB was not constant across the whole RoB latent trait, the total information curves (TIC) were used to verify where in the RoB latent trait there is more precision (i.e., amount of information). The utility of TIC is to inspect where, across the RoB latent trait, we can observe better precision (i.e., the amount of information captured) in specific RCTs. On the *X*-axis, the range of the RoB latent trait is presented in *z*-scores with a range from −3 to +3 and a mean of 0, where −3 is the highest RoB (lowest in terms of study quality) and +3 is the lowest RoB (highest in terms of study quality). On the *Y*-axis is the amount of information, which can range from 0 to infinity and thus has no maximum.

To evaluate Cochrane’s RoB reliability, we adopted the omega total (ω) (25, 26) because omega is an alternative to Cronbach’s alpha reliability coefficient. Omega total is used when the item set does not adhere to tau equivalence (i.e., the items does not contribute equally to the total scale score, not having, consequently, the same “weight” on how they are measuring RoB). Moreover, omega is used when the RoB items are not on a continuous scale, are not normally distributed, and the errors of the items (i.e., residual variance) may covary (i.e., the errors are correlated), as such, with the meta-analyses included in this study. Although Cronbach’s alpha presents this unrealistic assumptions (27, 28), to use both, unidimensionality is a requisite (29).

Omega total (ω) is a composite reliability index for congeneric scales (tests where the set of items are loaded onto a single factor) and is closely related to Cronbach’s alpha. Omega total assesses reliability *via* a ratio of the variability explained by items to the total variance of the entire scale (30, 31). This test information function arises from item response theory and can be considered a modern and powerful reliability estimation procedure when investigating the internal consistency of measurements (32). Also, according to Dunn et al. (29) “as the congeneric model allows item variances to vary (i.e., they are not assumed to be constant) it will not result in the lower bound estimations of reliability characteristic of alpha.”

The main advantages of omega over alpha can be summarized as follows: (1) Omega makes fewer and more realistic assumptions than alpha, (2) problems associated with inflation and attenuation of internal consistency estimation are far less likely, (3) employing “omega if item deleted” in a sample is more likely to reflect the true population estimates of reliability through the removal of a certain scale item, and (4) the calculation of omega alongside a confidence interval reflects much closer the variability in the estimation process, providing a more accurate degree of confidence in the consistency of scale administration (29).

The reliability measures are nearly always reported as point estimates, and there is no clear cutoff point. Bland and Altman (33) suggest that “a Cronbach’s alpha reliability coefficient greater than 0.7 is quite sufficient in many cases.” Kottner et al. (34) indicate that “values of 0.60, 0.70, or 0.80 are often used as the minimum standards for reliability coefficients in research, however, when applied in clinical practice, if individual and important decisions are made on the basis of reliability estimates, values should be at least 0.90 or 0.95.” As omega total (ω) has a similar interpretation to alpha, Rodriguez et al. (35) state that “a value higher than 0.8 indicates a sufficient relationship between the latent variable and item scores.”

Intrinsically linked with the reporting of alpha and omega as a point estimate, is the use of a cutoff heuristic, thought to reflect the crucial stage at which a scale possesses good or poor internal consistency (29). The heuristic in reliability reporting is based on work by Nunnally (36) who states that “what a satisfactory level of reliability is depends on how a measure is being used. In the early stages of research […] one saves time and energy by working with instruments that have only modest reliability, for which purpose reliabilities of 0.70 or higher will suffice […]. In contrast to the standards in basic research, in many applied settings a reliability of 0.80 is not nearly high enough. In basic research, the concern is with the size of correlations and with the differences in means for different experimental treatments, for which purposes a reliability of 0.80 for the different measures is adequate. In many applied problems, a great deal hinges on the exact score made by a person on a test […]. In such instances it is frightening to think that any measurement error is permitted. […]. In those applied settings where important decisions are made with respect to specific test scores, a reliability of 0.90 is the minimum that should be tolerated, and a reliability of 0.95 should be considered the desirable standard” [(36), pp. 245–246].

## Results

Initially, a 1-factor solution with nine items was tested, but the model was not admissible because the PAC item contained less than two categories (i.e., only the *high RoB* category was endorsed). Excluding PAC and rerunning the model with eight items returned a statistical problem because the minimum covariance coverage was not fulfilled. This error was due to problems in estimating correlation between two pairs of items: (a) BM and AC and (b) BM and OB. To keep the majority of the originally proposed items in the model, only the item, BM, was excluded. Rerunning the model with seven indicators resulted in a new inadmissible solution involving the AC item, and so AC was excluded. Thus, three items were excluded from the original set due to statistical problems related to the model’s convergence and admissibility. The model becomes estimable with 1-factor and 6 items, however, without appropriate fit indices [χ^2^(9) = 11.325, *p* = 0.254; CFI = 0.918; TLI = 0.864, RMSEA = 0.052 (90% confidence interval = 0.000–0.134, Cfit = 0.424); WRMR = 0.689], and with TLI being below the suggested cutoff.

The correlation between the RSG and the RoB attribute was not relevant (i.e., it was close to 0) and the factor loading was statistically insignificant (λ = 0.043, *p* = 0.692), indicating that RSG is poorly related to the RoB factor. Thus, RSG was excluded from the model. By removing the RSG indicator, the model with 1-factor and 5 items presented excellent fit indices, as follows: χ^2^(5) = 5.093, *p* = 0.4047; CFI = 0.997; TLI = 0.994, RMSEA = 0.014 (90% confidence interval = 0.000–0.145, Cfit = 0.536); WRMR = 0.689.

**Figure 1:**
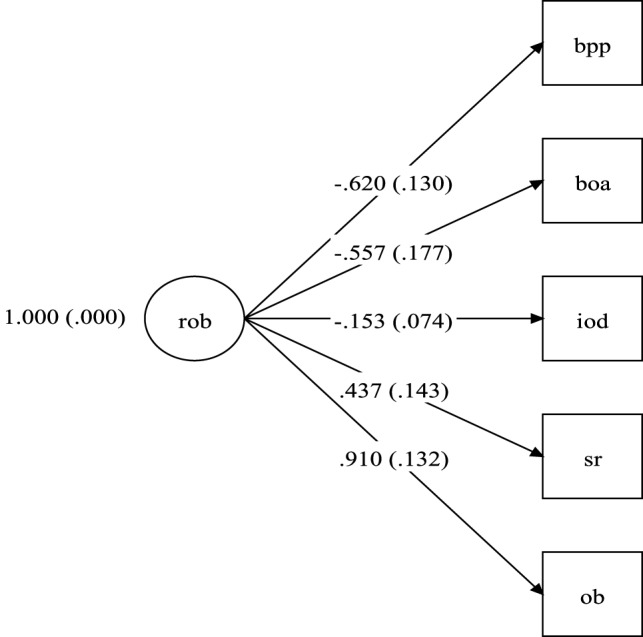
Risk of bias model. rob, risk of bias; bpp, blinding of participants and personnel (performance bias); boa, blind of outcome assessment (detection bias); iod, imcomplete outcome data (attrition bias); sr, selective reporting (reporting bias); ob, other bias. Note: numbers on left side of parentheses are the factor loadings and within the parentheses their respective SEs.

Figure 1 shows the diagram with the RoB model with excellent fit, made up of five indicators. As can be seen in Figure 1, the best item (the item most related to the RoB factor) is the *Other Bias* (λ = 0.910, *p* < 0.001). Negative factor loadings indicate that the items were inversely correlated to the RoB factor. The first two indictors, *blinding of participants and personnel* and *blind of outcome assessment* had moderate and statistically significant correlations with the RoB factor, indicating methodological strengths of the RCTs.

Therefore, the final model has excellent fit indices and factor loadings that vary from 0.153 to 0.910, with “*other bias*” exhibiting the highest factor loading. However, the amount of explained variance (i.e., *R*^2^) of the majority items in relation to the latent attribute (RoB) is small, as shown in Table 2.

**Table 2:** *R*^2^ and residual variances (measurement error) by item.

Items	*R*^2^	Residual variance
BPP	0.384	0.616
BOA	0.310	0.690
IOD	0.023	0.977
SR	0.191	0.809
OB	0.828	0.172

*BPP, blinding of participants and personnel (performance bias); BOA, blinding of outcome assessment (detection bias); IOD, incomplete outcome data (attrition bias); SR, selective reporting (reporting bias); OB, other bias*.

**Figure 2:**
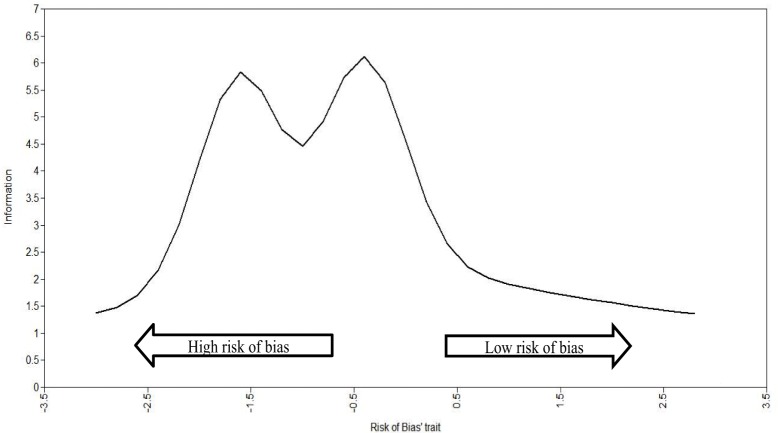
Total information curve of risk of bias factor.

Figure 2 shows the total curve of information for the RoB trait. It shows that the five items provide more precise information (i.e., higher amount of precision) in the lowest to middle spectrum of latent trait (i.e., *z*-score around −1.5 and −0.5). This means that the RoB latent trait was able to capture a greater amount of information in the RCTs with a low to medium amount RoB (the negative part of the spectrum). This means that the latent trait (RoB) captured by the RoB factor is more suitable for evaluating ASD intervention trials with a low RoB (it means lowest in terms of study quality).

Finally, the omega for the five indicators was 0.687 (95% CI: 0.613–0.761).

## Discussion

Based on CFA, we found that the five risks of bias items in the context of RCTs for ASD have construct validity returning excellent fit indices under a unidimensional model. The initial model with nine and seven items was not adequate to assess RoB for ASD intervention trials. For this study, only a unidimensional model was tested because there is no clear theory from the original descriptions of RoB tools provided by the Cochrane Collaboration (6) to support alternative configurations (e.g., seven indicators grouped into two factors).

It is important to note that, although the model fit indices showed strong values, the majority of the items had a greater amount of measurement error/residual variance than common variance, which raises an important issue of how reliably the items capture a unidimensional RoB factor as proposed by Cochrane. CFA includes the modeling of the measurement error associated with each indicator (latent variables regressed on the items with ε inside the ovals), and measurement error is what establishes the reliability of each item (37).

In terms of reliability, only one item, “*other bias*,” had high factor loading, low measurement error, and low residual variance. Four items had high measurement error and high residual variance and average factor loading. The item “*incomplete outcome data*” had very low factor loading and high measurement error and residual variance. This high degree of measurement error/residual variance across the majority of the items resulted in an omega total of 0.687 for the scale, indicating that the test is essentially 1% point below what is described above by Nunnally (36). Even with an upper confidence interval that is within what can be considered “acceptable,” the test indicates a less than moderate relationship between the latent variable and item scores and consequently a “low” reliability of test score.

Still, the item “*incomplete outcome data*” exhibited factor loadings below 0.4 and the item “*selective report*” was close to 0.4. According to Nunnally (38), “it is easy to overinterpret the meaning of small factor loadings, e.g., those below 0.40.” Therefore, among those remaining five items, only three had factor loadings higher than this cutoff. However, when we focused on the residual variance, the “other bias” item was the only item that exhibited more reliable variance. Consequently, this is reflected in the omega as a generally reliable measure of the set of items.

According to Brown (19), pp. 135–136, “… Even if a model is very successful at reproducing the observed relationships in the input matrix, this does not ensure that the latent variables are substantively interrelated or account for meaningful variance in the indicators. Thus, it is just as important to consider the size of the model’s parameter estimates as it is to consider the model’s goodness of fit in determining the acceptability of the solution.” This means that, for the model presented in this study, the latent factor is not substantively accounting for meaningful variance in the majority of the proposed RoB items.

In terms of precision, the set of items most reliable to measure RCT studies are the ones that have higher RoB (shown in Figure 2 by the studies which fall on the left side of the graph). These studies are of lower quality as they have a higher RoB.

Finally, although the items from our model show better indices, the results of this study were consistent with those of Rodrigues-Tartari et al. (8), who found that the RoB model for RCTs for trials of methylphenidate for children and adolescents with ADHD have poor reliability. Therefore, the Cochrane RoB tool is insufficient when applied to ASD clinical trials. As such, better indicators to capture RoB among intervention for ASD are needed.

## Author Contributions

All authors have made equal substantial contributions to all of the following: (1) the conception and design of the study, or acquisition of data, or analysis and interpretation of data, (2); drafting the article or revising it critically for important intellectual content; (3) final approval of the version to be submitted; and (4) agreement to be accountable for all aspects of the work in ensuring that questions related to the accuracy or integrity of any part of the work are appropriately investigated and resolved.

## Conflict of Interest Statement

The authors declare that the research was conducted in the absence of any commercial or financial relationships that could be construed as a potential conflict of interest.
